# Radio frequency sputtering of self-sanitizing material on NiTi archwires

**DOI:** 10.2340/biid.v12.45035

**Published:** 2025-12-11

**Authors:** Mahmood Naser, Emad Al-Hassani, Fatima Al-Hassani

**Affiliations:** a Department of Materials Engineering, Faculty of Engineering, University of Kufa, Iraq; b College of Materials Engineering, University of Technology, Iraq

**Keywords:** Archwire, coating, radio frequency, roughness, nanoparticles

## Abstract

**Objective:**

The objective of this study was to coat orthodontic nickel titanium (NiTi) archwires with nano-particles (NP) of silver (Ag) combined with nano polytetrafluoroethylene (PTFE) to produce a smooth antimicrobial nanocomposite layer by using a radio frequency (RF) sputtering process and to evaluate the coated surfaces in terms of morphology, nano-roughness, adhesion strength, hardness, and antimicrobial activity.

**Materials and methods:**

Super-elastic NiTi archwires (diameter = 0.4 mm, length = 160 mm) were surface cleaned and sterilized prior to the RF sputtering, using a mixture of nano Ag powder (20 nm; purity > 99.95%) and PTFE powder (25 nm; purity > 99.95%). X-ray diffraction apparatus (XRD), flex atomic force microscopy (AFM) and field emission scanning electron microscopy (FESEM) were used to characterize the morphology and nano roughness of the coated archwires. *Lactobacillus acidophilus (L. acidophilus)* and *Streptococcus mutans (S. mutans)* were selected to evaluate the antimicrobial activity.

**Results:**

A uniform and homogeneous nanocomposite coating was obtained without agglomeration. Surface roughness values decreased with increasing sputtering time, while the coated samples exhibited excellent antibacterial activity against both bacterial strains. AFM analysis demonstrated that sputtering time strongly influenced adhesion resistance, hardness, and coating stability, and the antibacterial activity was highly effective against both *L. acidophilus* and *S. mutans*.

**Conclusion:**

The sputtering time of 30 min gave a smooth coating layer on the surface of NiTi archwire with strong antimicrobial resistance that offers significant potential for dental applications.

## Introduction

In orthodontics, the most important concerns are the microbial accumulation around the archwires which have an undesirable impact on the health of the surrounding tissues [1]. The human body presents a challenging environment for metallic biomaterials, with the oral cavity serving as a critical entry point. Here, the variations in intraoral conditions are both frequent and intricate, resulting in a distinctive medium that promotes corrosion [2]. The oral environment serves as a prime setting for the unavoidable growth of microorganisms. To date, more than 700 bacterial species have been documented, alongside a variety of fungi and viruses. The oral microbiota has developed alongside humans in a mutually beneficial or even symbiotic relationship: The host offers optimal physiochemical and nutritional environments, while microorganisms, particularly bacteria, fulfill crucial physiological functions such as digestion, differentiation of oral mucosa cells, and defense against external pathogens [3–5].

A metal archwire used in orthodontics, such as nickel-titanium (NiTi), possesses exceptional superelasticity, resistance to corrosion, thermal shape memory and biocompatibility. In addition, the increasing number of adult orthodontic patients has resulted in an increase in the need for more cosmetic orthodontic appliances. This demand has been observed in the development of esthetic wires made of polymer, metallic arch wires covered with polymer materials, and wires that have been gold-plated [6]. Altering the surface characteristics of orthodontic components presents a viable approach to enhance their functionalities, particularly in inhibiting the development of bacterial biofilms. These alterations can be achieved through the incorporation of supplementary layers that include substances potentially exhibiting antibacterial properties [7]. The large surface area of the antimicrobial nanoparticles facilitates their interaction with bacterial cells of negative charge to a significant extent leading to enhanced antimicrobial activity. Moreover, antimicrobial nanoparticles added to polymers or coated onto surfaces of biomaterials were found to exhibit superior antimicrobial characteristics in the oral cavity [8, 9].

The installation of a fixed orthodontic appliance in patients often leads to a notable deterioration in oral health and hygiene. This decline is generally linked to the discomfort experienced as teeth shift into their new positions, along with the challenges of maintaining proper oral cleanliness due to the presence of numerous irregular surfaces [10]. Consequently, the application of antimicrobial agents, such as particular nanoparticles, to orthodontic devices warrants exploration as a potential method for preventing adherence and microbial colonization [11]. Nanoparticles made of silver, often known as silver (Ag) NPs, are among the nanoparticles that are investigated most frequently, as a result of the high aspect ratio (surface-to-volume ratio) of these substances, along with their low cost, cytotoxicity, immunological response, and antibacterial action even at low concentrations. These nanoparticles have become a more desirable option for use in biomedical applications as a result of the extensive variety of potential that Ag-NPs possess [12, 13]. It is well known that polytetrafluoroethylene (PTFE) exhibits intrinsic non-adhesive characteristics as a result of its minimal surface energy. The incorporation of PTFE nanoparticles with the Ag NPs by radio frequency (RF) sputtering can produce a coating layer with favorable properties, like bacterial resistance, scaling resistance and smooth surface layer (affects surface roughness) [14, 15]. It was reported that the Ag/PTFE sputtered composite coatings demonstrated a high antibacterial efficacy, achieving removal rates of 99.99% for *Staphylococcus aureus* [16, 17]. Moreover, the Ag/PTFE sputtered composite coating was capable of releasing antibacterial Ag+ ions in a sustained manner, which effectively inhibited bacterial growth and reduced biofilm formation by approximately 50% after 7 days [18].

The motivation for this study is the clinical need to reduce microbial adhesion and biofilm formation on archwires, which contribute to enamel demineralization, gingival disease, and oral infections. The problem addressed is the lack of coatings that can simultaneously improve surface properties and enhance antibacterial performance. Previous studies have often investigated coatings based on either Ag, owing to its strong antimicrobial properties, or polymers such as PTFE, due to their low-friction characteristics, but rarely their combination. This has left a gap in understanding how Ag/PTFE nanocomposite coatings behave when applied to NiTi archwires. It was hypothesized that integrating Ag nanoparticles with PTFE in a nanocomposite coating, deposited by RF sputtering, would yield smoother surfaces with lower adhesion and enhanced antibacterial activity compared to single-component coatings. This assumption is supported by prior findings that Ag provides potent antibacterial effects, while PTFE reduces surface energy and friction when used as a coating material [19, 20]. In this study, Ag and PTFE were sputtered under different deposition times, and the resulting surfaces were systematically evaluated in terms of morphology, nano-roughness, adhesion strength, hardness, and antibacterial activity against *Lactobacillus acidophilus* and *Streptococcus mutans*.

## Materials and methods

The primary materials used in this work and their properties are listed in Table 1.

**Table 1:** Description of the main materials utilized in this study.

No.	Material	Specifications	Purpose
1	Super-elastic NiTi archwires (NTW)	Diameter: 0.4 mm Length: 160 mm Company: Dentaurum, Germany	Substrate
2	Silver (Ag) powder	Size: 20 nm, Purity: > 99.95% Density: 0.5 g/cm^3^ Company: Yujiang Chemical, China	Prepare RF coating
3	Polytetrafluoroethylene (PTFE) powder	Size: 25 nm, Purity: > 99.95% Density: 2.2 g/cm^3^ Company: Yujiang Chemical, China	Prepare RF coating

### Target preparation for RF sputtering process

The RF target was made from Ag/PTFE nano powders weighing 100 g. The powders were mixed together in four tanks mixer (MTI, USA) for 3 h at 100 rpm and then compressed by using a hydraulic press (Mega, PRDE (30T/50T), Spain). The mixed powder was compacted under a pressure of 500 MPa for 5 min, resulting in a disk specimen of 60 mm diameter and 5 mm thickness.

### Archwire preparation for RF sputtering process

NiTi archwires (NTWs) were cleaned to eliminate any surface contamination by using an ultrasonic cleaner bath (MTI Corporation, USA) containing deionized water for 10 min. After drying, the NTWs were sterilized in an ultraviolet light cabinet (Analytic, Germany) for 30 min [21].

### RF sputtering process

The RF target was affixed to the cathode, while the archwires were positioned on the anode, oriented towards the target holder, which was situated 60 mm away from the target within the RF sputtering apparatus (Barez Afarin industry, Iran). The sputtering chamber underwent initial evacuation to achieve a pressure of 6 × 10^−4^ Pa prior to the presenting of high-purity (99.9999%) working argon (Ar) gas with 20 mL min^−1^ flow rate. Throughout the sputtering process, the holder of the substrate was subjected to 20 RPM rotational speed. The Ag/PTFE layer was sputtered at 0.6 Pa gas pressure and utilizing a sputtering power of 60 W. Three sputtering durations were tested: 10, 20, and 30 min. All samples were prepared under ambient conditions.

### Characterization techniques

X-ray diffraction was performed to define the presence of phases and crystallographic properties of the raw materials, the coated and the uncoated samples by using X-Ray diffractometer (Chongqing Drawell, China) with a nickel filter and copper generator. The speed of scanning was adjusted to 6 deg.min−1 and the diffraction angle (2θ^o^) range was (10^o^–80^o^).

Surface topography, surface nanoroughness (Ra), adhesion strength and hardness, and particle size distribution of the coated and uncoated NTW were investigated by using a flex atomic force microscopy (AFM) with interchangeable cantilever holder (Nanosurf, Switzerland).

The surface morphology, size and distribution of nanoparticles and the coating layer thickness from the cross-section microstructure were investigated by using field emission scanning electron microscopy (FESEM) (TESCAN MIRA3, France). The elemental composition of the samples was examined using energy dispersive X-ray spectroscopy (EDX) in conjunction with the FESEM.

### Antibacterial assay

Antibacterial activities of the surface-modified orthodontic wires were demonstrated against *Lactobacillus acidophilus (L. acidophilus)* and *Streptococcus mutans (S. mutans)* bacterial strains. The agar well diffusion technique was used to investigate the antimicrobial effect of the uncoated and coated NTWs. The prepared cultures were spread on petri dishes, and then the samples were placed in an incubator. After incubation for 24 h and at 37°C, the inhibition zone size was measured using a ruler.

### Statistical analysis

Descriptive statistics was used in this study for the nano roughness and antimicrobial data which were organized, categorized and transferred into a computerized database structure using SPSS program and it includes Minimum (Min), Maximum (Max), Mean (M), standard deviation (SD), standard error (SE), and data were screened for normal distribution and homogeneity using Shapiro-Wilk and Levene’s tests respectively. These tests were employed to either accept or reject the statistical hypothesis, establishing a confidence interval of 95%. The significance level was deemed significant when the *p*-value fell below 0.05. One-way analysis of variance (ANOVA) was used to compare Ra among the four groups. When the ANOVA indicated significant differences (*p* < 0.05), pairwise comparisons were performed using Tukey’s honestly significant difference (HSD) post‑hoc test. Significance was set at *p* < 0.05. All analyses were conducted using IBM SPSS Statistics (Version 31.0.0.0, IBM Corp., USA).

## Results

### Surface characterization and morphology

**Figure 1:**
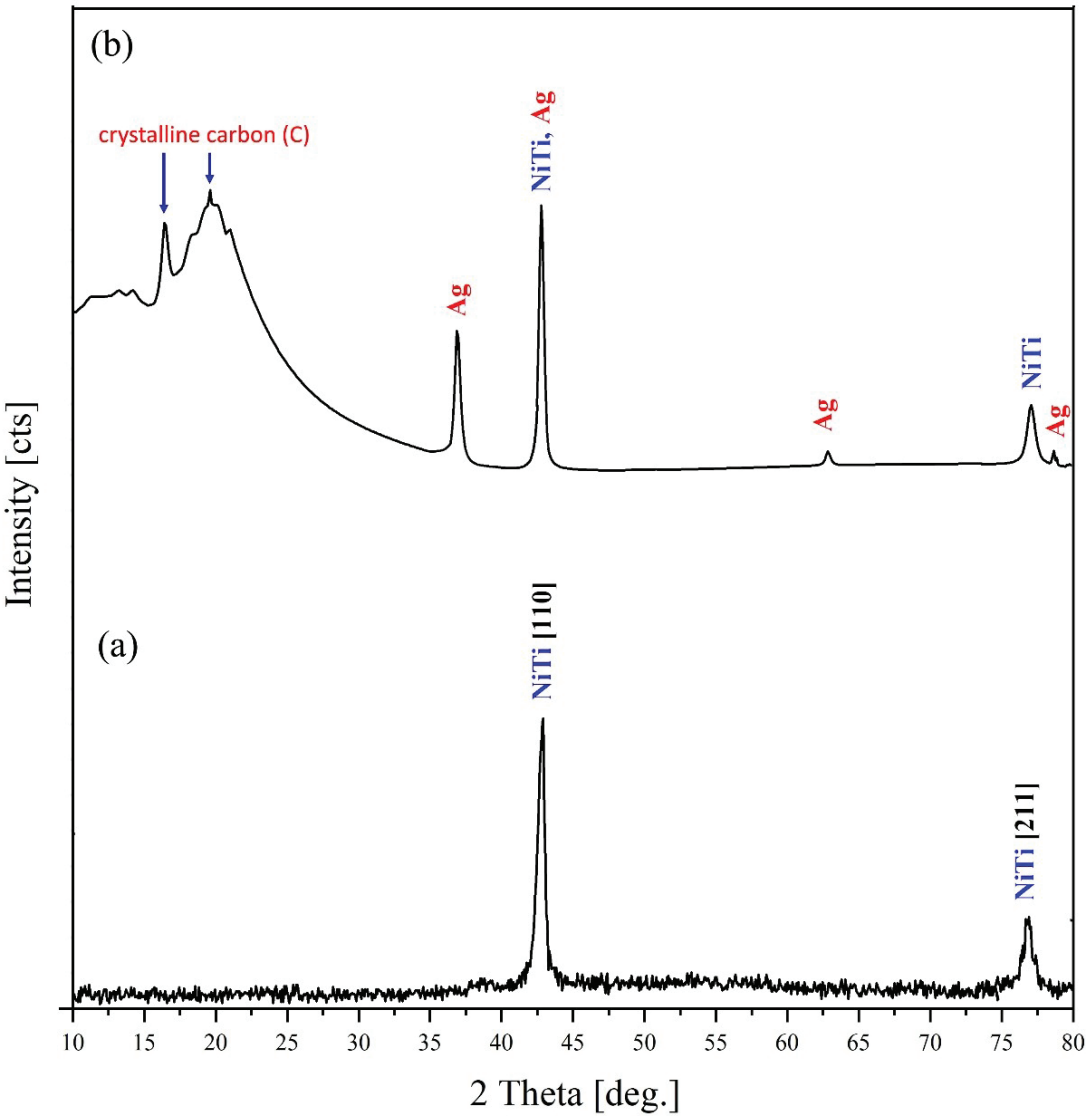
X-ray diffraction pattern of uncoated (A) and (B) silver/polytetrafluoroethylene-coated NiTi archwires.

The X-ray diffraction (XRD) patterns of the uncoated NTW (Figure 1A) showed two primary diffraction peaks corresponding to the NiTi alloy phase (110) and (211) respectively, while the Ag/PTFE coated NTW (Figure 1B) exhibited diffraction peaks of both Ag and PTFE from the thin coated layer. The diffraction peaks at 2θ value of 37.3^o^ considered the primary peak of Ag which is correspond to the (111) plane that confirm the presence of Ag in addition to the two other weak peaks at 2θ value of 64.4^o^ and 77.3^o^, which corresponds to (220) and (311) planes of metallic silver having face center cube (FCC) crystal symmetry and all the peaks corresponded to the standard Joint Committee on Powder Diffraction Standards (JCPDS) card No. 04-0783 of cubic AgNPs [22]. Finally, two diffraction peaks were observed at 2θ values of 16.8° and 18.3°, which correspond to the crystallinecarbon of PTFE phase [23]. From the XRD results, it can be seen that a Ag/PTFE coating layer was coated on NTW.

**Figure 2:**
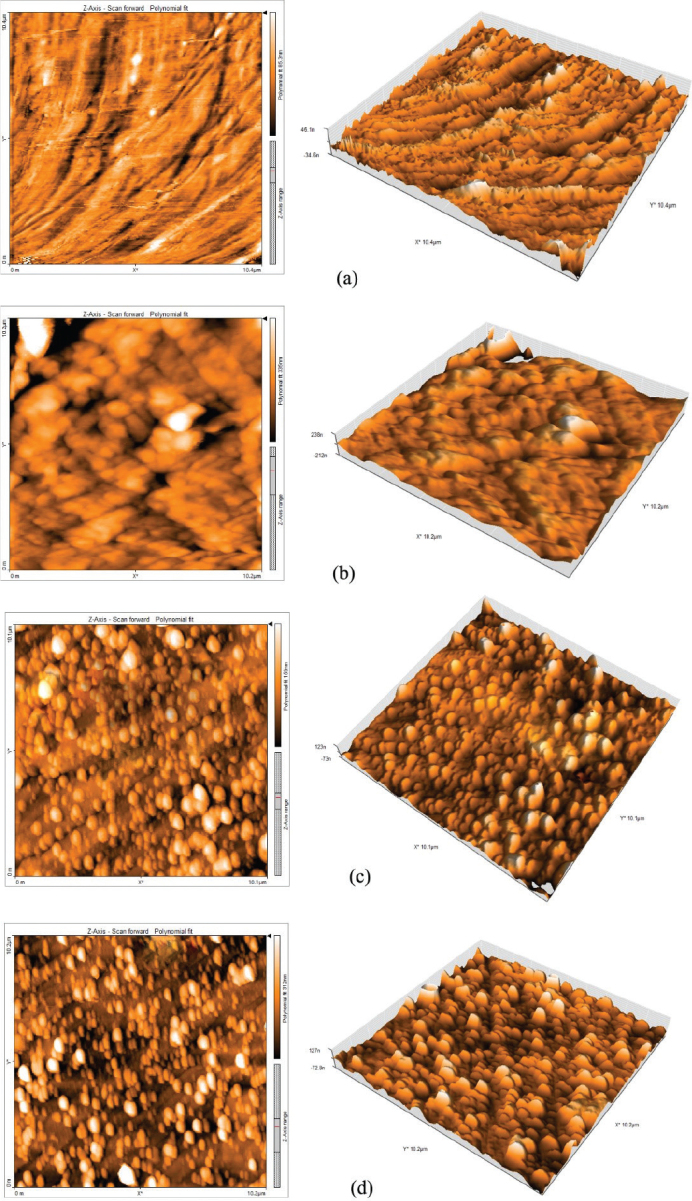
Surface morphology of uncoated NiTi archwires (NTW) (A) and silver/polytetrafluoroethylene coated NTW at different sputtering times (B) 10 min, (C) 20 min, and (D) 30 min.

Two and three-dimensional surface topographies of both coated and uncoated NTW at varying sputtering times are shown in Figure 2. The prominent microstructure on the surface varied in shape depending on the sputtering time. In Figure 2B, the brown area indicates the recessed part, while the area in white represent the raised part. The color transition in (Figure 2C and D) was slight, representing the protrusion height without any clear differences. The larger white parts in the Figures were assumed to the structure of the sputtered island. The results of the surface roughness measurements are shown in Table 2.

**Table 2:** Surface roughness (nm) of uncoated and silver/polytetrafluoroethylene coated NiTi archwires.

Sample symbol	Sample condition	Sputtering time (min)	*N*	Min	Max	Mean	SD	SE	Shapiro- Wilk test *p*
NT	Uncoated NTW	0	5	50.2	64.5	57.06	5.85	2.62	0.654
a	Coated NTW	10	5	70.7	80.1	75.46	3.52	1.58	0.999
b	Coated NTW	20	5	39.2	50.0	44.68	4.83	2.16	0.425
c	Coated NTW	30	5	18.7	22.6	20.80	1.45	0.65	0.975

NTW: NiTi archwires; NT: uncoated wire.

The Shapiro-Wilk test found the data to be normally distributed, and Levene’s test found the variances to be homogeneous. One-way ANOVA showed a significant effect of sputtering time on surface roughness (Ra): F(3, 16) = 145.88, *p* = 7.8678 ×10^-12^. The F statistic and corresponding *p*-value indicate a highly significant difference among the sputtering time groups (*p* < 0.05). Tukey’s HSD (Table 3) indicated that all pairwise comparisons between groups were statistically significant (*p* < 0.05). The uncoated wire (NT) exhibited moderate roughness, the 10 min sputtered group (1) showed the highest Ra, whereas increasing sputtering time to 20 min (2) and 30 min (3) significantly reduced roughness, with the smoothest surface observed at 30 min.

**Table 3:** Tukey post-hoc results.

Comparison	Mean difference	*p*	Significance
NT versus A	10.01	0.0003	Yes
NT versus B	-18.04	< 0.0001	Yes
NT versus C	-44.43	< 0.0001	Yes
A versus B	-28.05	< 0.0001	Yes
A versus C	-54.44	< 0.0001	Yes
B versus C	-26.39	< 0.0001	Yes

Note: *p*-values indicate significance levels; comparisons with *p* < 0.05 were considered significant.

**Figure 3:**
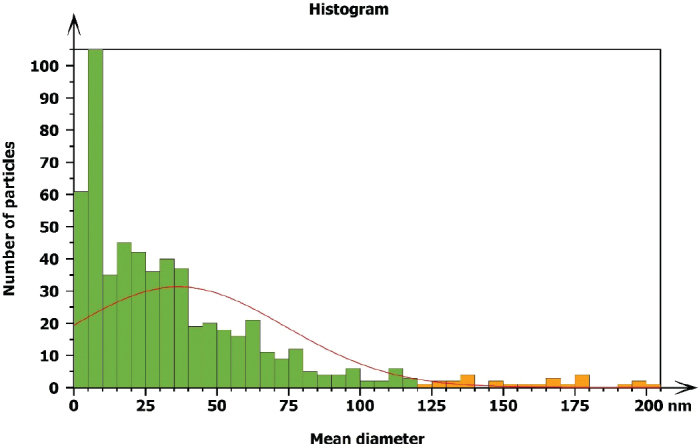
Histogram for the silver/polytetrafluoroethylene nanoparticles coated on the NiTi archwires at 30 min, explaining the percentage of nanoparticle size and its distribution.

The particle size distribution of Ag/PTFE nanoparticles are shown in Figure 3. The average particle size of 48.11 nm is located within the nanoscale level (1~100 nm) [24].

**Figure 4:**
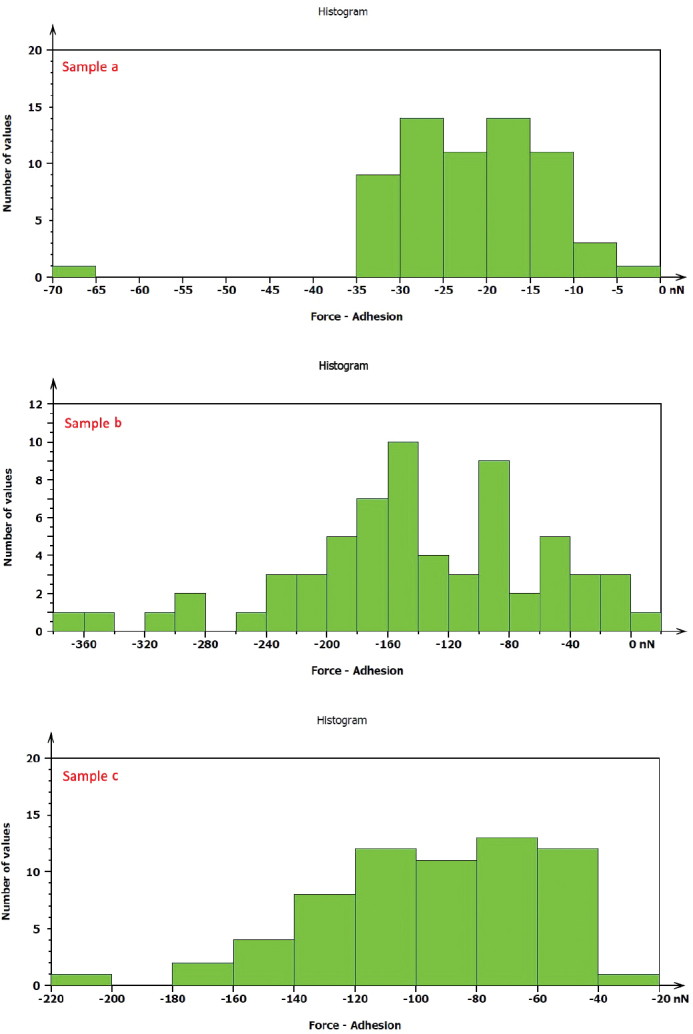
Adhesion strength/pull off of different coated NiTi archwires.

AFM revealed distinct differences in adhesion among the sputtered Ag/PTFE nanocomposite coatings on NiTi wires. Figure 4 shows the adhesion forces/pull off obtained for the three sputtered coatings. Sample a (10 min sputtering) exhibited the lowest adhesion (−12.22 nN), indicating that shorter sputtering time produced a thinner, less consolidated surface with reduced interfacial forces. Sample b (20 min sputtering) presented moderate adhesion (−25.85 nN), while Sample c (30 min sputtering) showed an increased adhesion strength. The overall ranking of adhesion resistance was therefore: a (10 min) > b (20 min) > c (30 min).

Indentation results (Table 4) also demonstrated a strong dependence on sputtering time. Sample a (10 min) displayed the lowest modulus (~0.17 GPa), reflecting a relatively soft and less consolidated layer. Increasing sputtering to 20 min (Sample b) enhanced the modulus (~0.3 GPa), suggesting a thicker and more stable film. Sample c (30 min) provided intermediate values (~0.4 GPa) with good fitting, indicating that extended sputtering improved coating consolidation and durability, with an increase in adhesion.

**Table 4:** Effect of sputtering time on adhesion, modulus, and stability of silver/polytetrafluoroethylene coated NiTi archwires.

Sample	Sputtering time (min)	Adhesion force (nN)	Indentation modulus (GPa)	Stability & durability (qualitative)
a	10	−12.22	~0.177	Low stability; prone to wear/delamination
b	20	−25.85	~0.3	Good stability; balanced adhesion and hardness
c	30	−34.72	~0.4	Best balance; durable with increased adhesion

Note: It should be noted that the adhesion (pull-off) forces obtained by AFM are expressed as negative values, representing the tensile force required to detach the cantilever tip from the coating surface. Therefore, a more negative value (greater absolute magnitude) indicates stronger adhesive interaction between the AFM tip and the surface.

**Figure 5:**
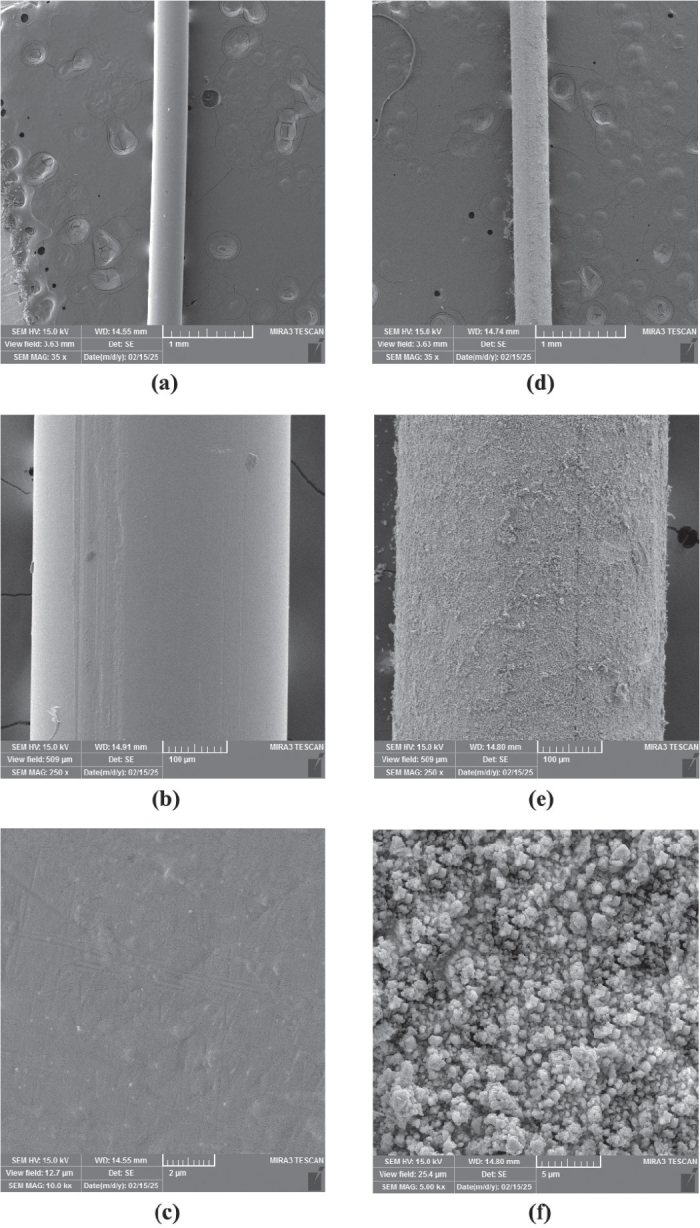
Field emission scanning electron microscopy images of the NiTi archwires (NTW) at different magnification powers for the (A, B, C) uncoated and (D, E, F) silver/polytetrafluoroethylene coated NTW at 30 min.

The FESEM images of NTWs at various magnifications (35X, 250X and 10KX) are shown in Figure 5. The images demonstrate a distinct disparity between the surface topography of the uncoated archwire segment and that of the Ag/PTFE coated segment. The Ag/PTFE nanoparticle-coated NTW segments revealed a uniform distribution of spherical morphology.

**Figure 6:**
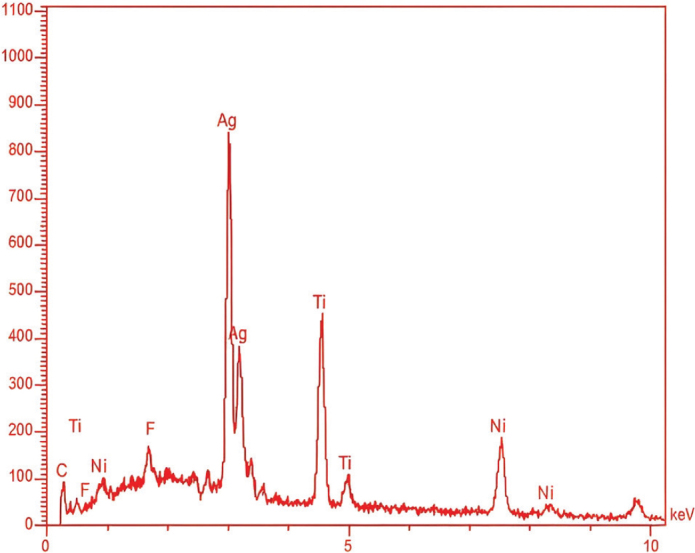
EDX spectroscopy charts for silver/polytetrafluoroethylene-coated NiTi archwires at 30 min.

**Figure 7:**
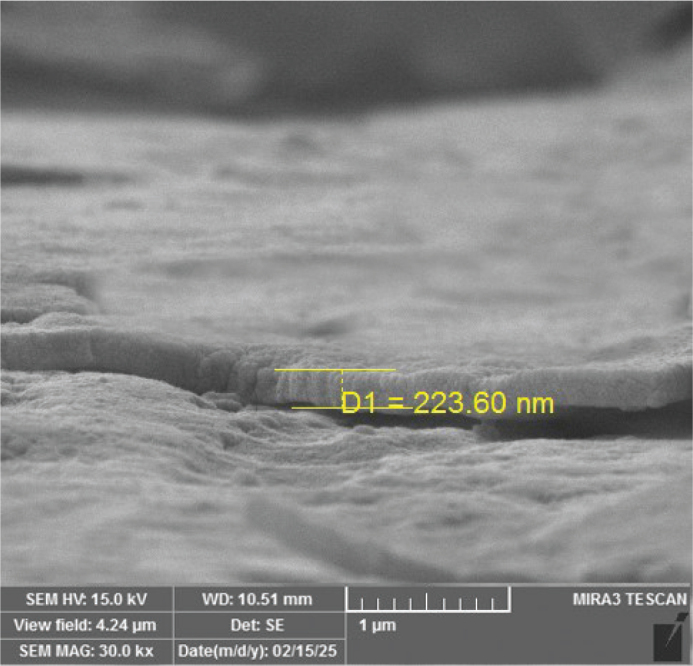
Cross-section microstructure of the silver/polytetrafluoroethylene coating layer.

**Figure 8:**
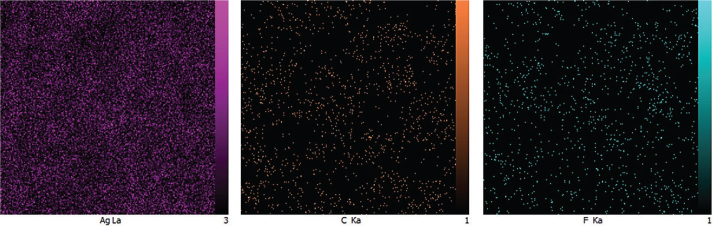
EDX mapping of the field emission scanning electron microscopy sample coated at 30 min.

The EDX results (Figure 6) obtained during the FESEM analysis revealed the weight percentages of various elements, including Ag, fluorine (F), and carbon (C) from the coated layer, along with nickel (Ni) and titanium (Ti) from the substrate. The presence of carbon and fluorine served as the basis for identifying PTFE. This demonstrates that the RF sputtering occurs as a thin layer capable of being penetrated by the EDX electrons (Figure 7). The EDX mapping in Figure 8 illustrates the distributions of Ag, C, and F within the coating layer.

## Antimicrobial assessment

**Figure 9:**
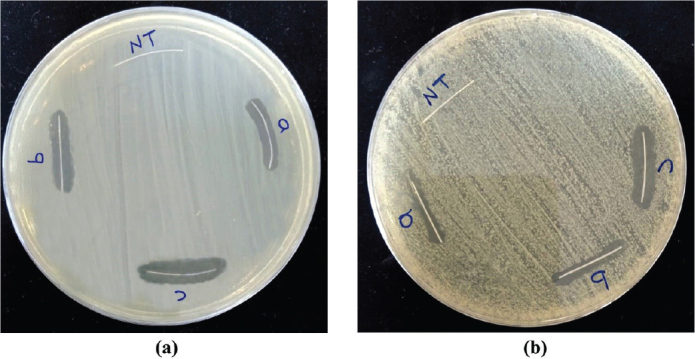
Antimicrobial assessment for the uncoated and coated NiTi archwires at different sputtering times for (A) S. mutans, (B) L. acidophilus.

The antimicrobial activity was illustrated by the formation of a clear inhibition zone around each NTW for both *S. mutans and L. acidophilus* while there was no microbial growth suppression of the uncoated NTW (Figure 8). The results of the inhibition zone measurements are shown in Table 5 and Figure 9.

**Table 5:** Inhibition zones (mm) for silver/polytetrafluoroethylene coated NiTi archwires in different microbial petri dishes.

Sample symbol	Sputtering time (min)	Microbial Species	*N*	Mean	SD
a	10	*S. mutans*	5	11	0.339
b	20		5	12.14	0.513
c	30		5	15.12	0.311
a	10	*L. acidophilus*	5	9.88	0.653
b	20		5	11.06	0.527
c	30		5	14.86	0.270

All the coated NTW exhibited an antibacterial behavior but in different activity. The Shapiro-Wilk test found the data to be normally distributed, and Levene’s test found the variances to be homogeneous. The antibacterial analysis showed consistent trends for both *S. mutans* and *L. acidophilus*, ANOVA revealed highly significant differences in inhibition zone diameters among sputtering times (*p* < 0.0001) for both species. According to Tukey’s test (Table 6), all coated groups (a, b, and c) exhibited significantly larger inhibition zones compared to the uncoated control (NT). For both bacteria, the 30 min sputtered sample (c) demonstrated the largest inhibition zone, followed by 20 min (b) and 10 min (a), indicating enhanced antibacterial activity with prolonged sputtering time.

**Table 6:** Tukey post-hoc results for S. mutans and L. acidophilus.

Comparison	Microbial species	Mean difference	*p*	Significance
A versus B	*S. mutans*	1.14	0.0004	Yes
A versus C		4.12	< 0.0001	Yes
A versus NT		-11.00	< 0.0001	Yes
B versus C		2.98	< 0.0001	Yes
B versus NT		-12.14	< 0.0001	Yes
C versus NT		-15.12	< 0.0001	Yes
A versus B	*L. acidophilus*	1.18	0.0032	Yes
A versus C		4.98	< 0.0001	Yes
A versus NT		-9.88	< 0.0001	Yes
B versus C		3.80	< 0.0001	Yes
B versus NT		-11.06	< 0.0001	Yes
C versus NT		-14.86	< 0.0001	Yes

## Discussion

The sputtered Ag/PTFE nanocomposite coatings produced on NiTi orthodontic wires exhibited significant improvements in surface morphology, adhesion, and antibacterial performance with increasing sputtering time. The standard peak intensities of the NTW XRD were lowered as a result of the incorporation and interaction between Ag and PTFE. AFM and FESEM analyses confirmed the formation of a smooth, dense, and uniform coating structure, particularly in the 30 min sputtered group. The reduction in surface roughness with extended sputtering time is attributed to improved film consolidation and uniform nanoparticle distribution reflecting the layer growth mechanism. This smoother morphology enhances coating adhesion to the NiTi substrate by reducing interfacial voids [25]. In addition, PTFE itself is known for its low surface energy and ability to form smooth, uniform layers. When deposited via RF sputtering, it can fill in micro-pores and irregularities on the substrate, resulting in a more compact and even surface morphology [26]. These findings agree with previous studies that reported improved nanocomposite film stability and durability under prolonged sputtering conditions [27]. While short deposition (10 min) resulted in a rougher, less consolidated layer and this can be attributed to the aggregation of the small tip structures into larger surface protrusions [28], these trends support the selection of 30 min sputtering when minimal surface roughness is required.

The mechanical characterization also revealed that higher sputtering durations enhanced the coating modulus and adhesion strength, indicating a more cohesive and stable film. Such structural uniformity plays a key role in the functional performance of coated orthodontic wires, as it prevents delamination during intraoral loading [29]. Moreover, the reduction in surface roughness was found to be inversely proportional to bacterial adhesion, suggesting that surface smoothness acts as a physical barrier against microbial colonization [30]. From a stability perspective, short sputtering (10 min) produced the lowest adhesion but also the weakest mechanical durability. Intermediate sputtering (20 min) improved both modulus and stability while maintaining low adhesion. Extended sputtering (30 min) yielded the most balanced performance, combining increased adhesion with reliable mechanical stiffness and long-term durability. The comparable results obtained for *S. mutans* and *L. acidophilus* indicate that the antibacterial mechanism of the Ag/PTFE nanocomposite coating is broadly effective against both Gram-positive bacterial species. The increasing inhibition with longer sputtering times is attributed to improved coating uniformity and Ag nanoparticle density, which enhances silver ion release and surface contact with bacterial membranes. The uncoated NTW showed no inhibition zones, confirming that antibacterial performance is directly related to the sputtered Ag/PTFE layer. Overall, the 30 min sputtered coating provided the most stable and efficient antibacterial response. These results are consistent with recent reports demonstrating that hybrid metallic–polymeric nanocomposites exhibit prolonged antimicrobial effectiveness and reduced cytotoxicity compared to single-phase coatings [31]. The enhanced surface quality and antibacterial stability achieved at 30 min sputtering suggest strong potential for clinical applications, particularly in maintaining long-term microbial resistance and minimizing frictional wear during orthodontic treatment.

## Conclusion

In this study, it was concluded that extending the sputtering time during RF deposition of Ag/PTFE nanocomposite coatings on NiTi orthodontic wires significantly improves coating uniformity, adhesion, and antibacterial properties. The 30 min sputtering duration yielded the most balanced performance, with optimal surface smoothness, mechanical durability, and bacterial inhibition against *S. mutans and L. acidophilus*. These findings highlight the potential of Ag/PTFE nanocomposite coatings as a promising surface modification for orthodontic applications, combining mechanical stability with long-term antimicrobial protection.

## Supplementary Material




