# Supplementary figures and images for: Radio frequency sputtering of self-sanitizing material on NiTi archwires
Source: Biomater Investig Dent. 2025 Dec 11;12:45035. doi: 10.2340/biid.v12.45035 (PMC12706999; doi:10.2340/biid.v12.45035)

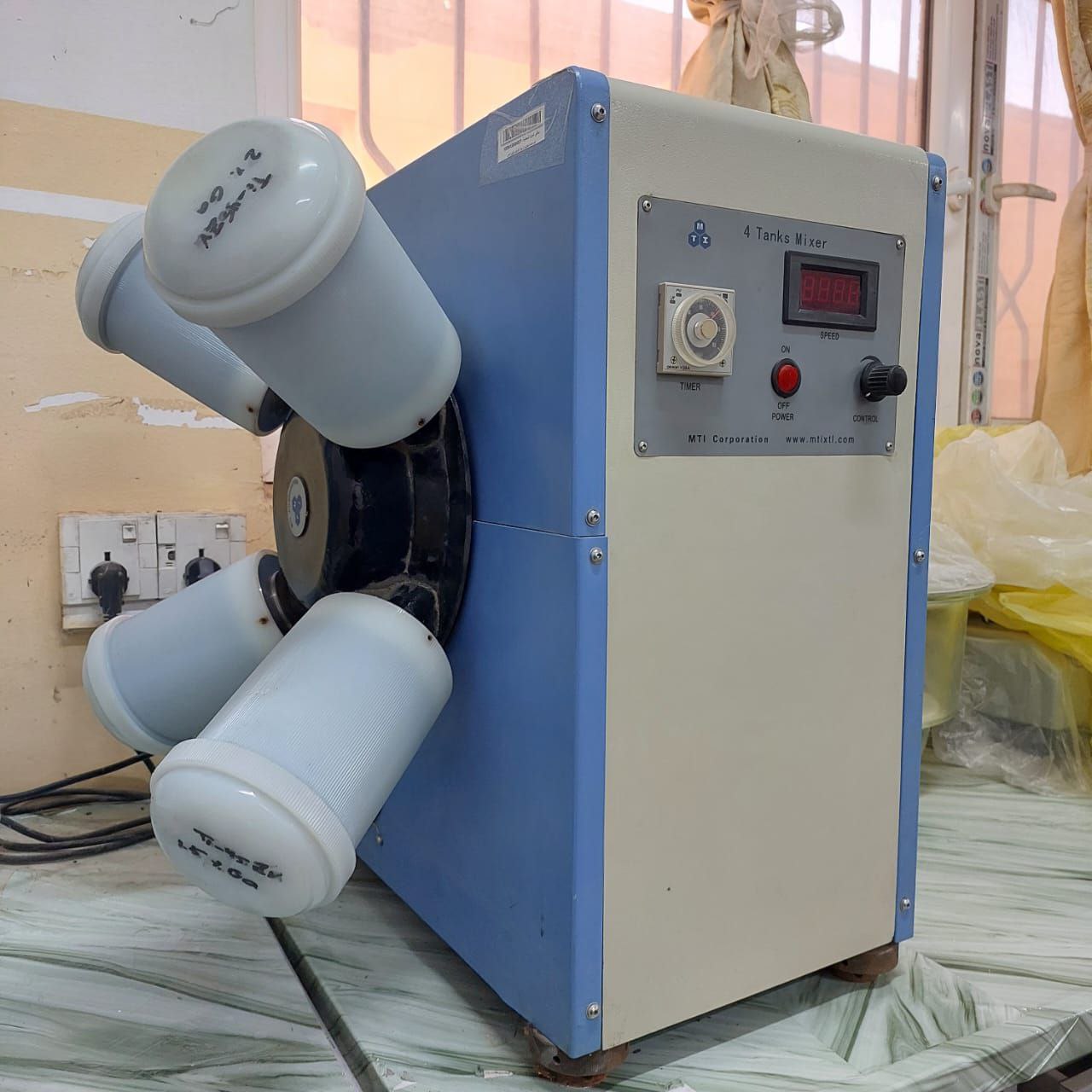

Supplement: Supplementary file 1 [file BIiD-12-45035-s1.jpg]

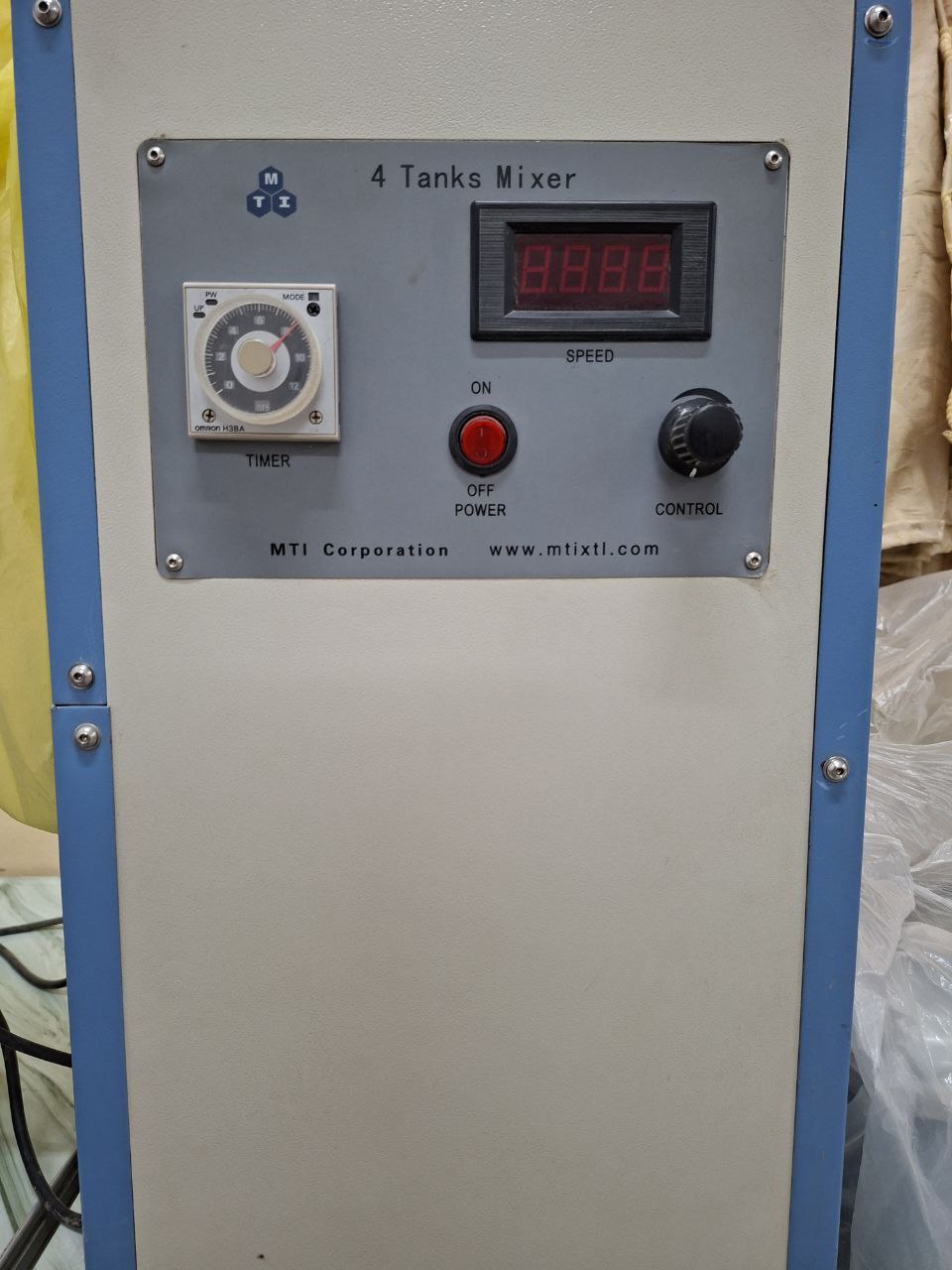

Supplement: Supplementary file 2 [file BIiD-12-45035-s2.jpg]
